# A Novel Outdoor Positioning Technique Using LTE Network Fingerprints

**DOI:** 10.3390/s20061691

**Published:** 2020-03-18

**Authors:** Da Li, Yingke Lei, Haichuan Zhang

**Affiliations:** 1 College of Electronic Engineering, National University of Defense Technology, Hefei 230000, China; leiyingke@163.com (Y.L.); zhanghai4258@163.com (H.Z.); 2 Science and Technology on Communication Information Security Control Laboratory, Jiaxing 314000, China

**Keywords:** fingerprint positioning, outdoor positioning, deep learning, LTE signals, transfer learning

## Abstract

In recent years, wireless-based fingerprint positioning has attracted increasing research attention owing to its position-related features and applications in the Internet of Things (IoT). In this paper, by leveraging long-term evolution (LTE) signals, a novel deep-learning-based fingerprint positioning approach is proposed to solve the problem of outdoor positioning. Considering the outstanding performance of deep learning in image classification, LTE signal measurements are converted into location grayscale images to form a fingerprint database. In order to deal with the instability of LTE signals, prevent the gradient dispersion problem, and increase the robustness of the proposed deep neural network (DNN), the following methods are adopted: First, cross-entropy is used as the loss function of the DNN. Second, the learning rate of the proposed DNN is dynamically adjusted. Third, this paper adopted several data enhancement techniques. To find the best positioning fingerprint and method, three types of fingerprint and five positioning models are compared. Finally, by using a deep residual network (Resnet) and transfer learning, a hierarchical structure training method is proposed. The proposed Resnet is used to train with the united fingerprint image database to obtain a positioning model called a coarse localizer. By using the prior knowledge of the pretrained Resnet, feed-forward neural network (FFNN)-based transfer learning is used to train with the united fingerprint database to obtain a better positioning model, called a fine localizer. The experimental results convincingly show that the proposed DNN can automatically learn the location features of LTE signals and achieve satisfactory positioning accuracy in outdoor environments.

## 1. Introduction

The explosion of outdoor location-based services (OLBSs) in the Internet of Things (IoT) has stimulated extensive research efforts in recent years [1]. Traditional OLBSs rely on satellite navigation positioning systems, such as the Global Positioning System (GPS), and they bring great benefits and convenience to many people. However, under the conditions of signal non-line-of-sight (NLOS) propagation, such as in crowded cities or during unfavorable weather, the positioning accuracy normally deteriorates greatly, which can cause a range of problems. Furthermore, GPS requires a huge amount of energy consumption [2]. The large-scale application of long-term evolution (LTE) signals and multiple low-energy consumption sensors equipped in devices such as smartphones have brought about a new method for outdoor positioning techniques [3].

Traditional wireless outdoor positioning technologies normally refer to measurement-based systems that infer the position of user equipment (UE) based on the signals of angle of arrival (AOA), time of arrival (TOA), time difference of arrival (TDOA), or hybrid methods [4]. However, these positioning technologies require additional devices, making these systems impractical. Additionally, in signal NLOS propagation, the performance of the positioning systems will be greatly reduced [5].

In contrast, wireless fingerprint positioning technology has received much attention owing to its simplicity and practicality with existing infrastructure and hardware. Many complex LTE signal cues are hidden in the surrounding environment, and the goal of wireless fingerprint-based localization is to discover these cues and use them effectively to determine UE positions [6]. Compared to satellite navigation positioning systems and other wireless positioning technologies, fingerprint-based positioning techniques have many merits. First, low-energy consumption sensors in UE require much less energy. Second, most of the fingerprint-based positioning technologies require no additional devices or infrastructure. Finally, fingerprint-based positioning can be achieved by using ubiquitous smartphones and signals from LTE base stations (BSs) [6,7]. Fingerprint positioning technology works in two phases: an offline training phase and an online matching phase [8]. During the training phase, signal measurements can be used, which are usually received signal strength indicator (RSSI), reference signal receiving power (RSRP), received signal receiving quality (RSRQ), or channel state information (CSI) [9], as the fingerprint. These measurements are collected at known locations in a database and regard the fingerprint database as the radio map. During the online matching phase, the locations are determined by comparing the real-time LTE signal measurements with the entire fingerprint database. The majority of fingerprint-based outdoor positioning relies on RSSI and RSRP as the signal measurements, owing to their easy accessibility at both the transmitter and receiver sides [10]. For instance, there are numerous LTE BSs and Wi-Fi access points (APs), and their coverage includes almost all urban areas. Additionally, RSSI and RSRP can be measured with just one smartphone. However, when using Wi-Fi signals for positioning, due to the numerous Wi-Fi APs, the data preprocessing will be labor-intensive and time-consuming, especially when measuring large areas. Additionally, it cannot be used in rural areas since most of these areas are not covered by Wi-Fi [11].

**Figure 1:**
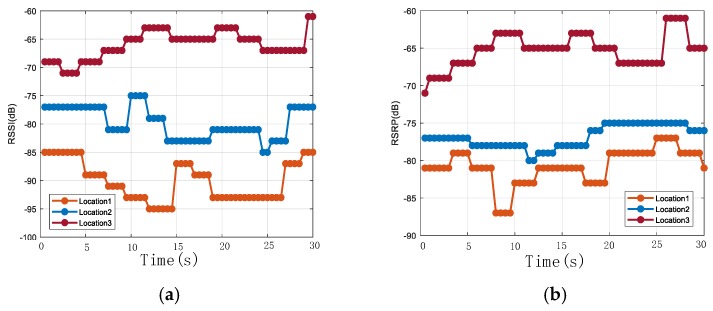
Signal fluctuation of (**a**) received signal strength indicators (RSSIs) and (**b**) reference signal receiving powers (RSRPs) at three fixed locations over time. Each graph contains 60 samples.

The first fingerprint positioning system relied on the k-nearest neighbors (KNN) algorithm to find the best matches from the fingerprint database [12]. Weighted k-nearest neighbors (WKNN) was proposed to enhance the robustness of the fingerprint positioning system [13]. Later, a generalized regression neural network (GRNN) [14] and support vector machine (SVM) [15] were introduced to target an accurate solution to the localization problem. However, the major problems in achieving highly precise positioning results come from the instability of wireless signals, such as attenuation by buildings, trees, or moving people [16]. Figure 1 shows the fluctuation of the LTE signal’s RSSI and RSRP from one LTE BS at three fixed positions over time. It shows that the measurements change frequently over a wide range. Therefore, it is necessary to collect more wireless signal samples in order to achieve highly precise positioning, especially when measuring a large-scale outdoor environment. Therefore, the main challenge lies in finding a reliable fingerprint and developing a good model to extract discriminative features from a large set of fluctuating wireless signals [6,17].

The aforementioned matching algorithms have unsatisfactory positioning accuracy owing to their limited learning ability. As it has developed, deep learning has shown excellent modeling ability in computer vision, speech recognition, pattern recognition, and so on [18]. It has been successfully proven that deep learning can beat other state-of-the-art technologies. Therefore, in this work, we investigated whether deep learning can provide a solution for large-scale outdoor positioning.

Considering the outstanding learning ability of deep learning in image classification, in this paper LTE signal measurements are converted into novel fingerprint grayscale images to represent the features of different positions. The fingerprint image dataset is used as the input of the proposed Resnet. Additionally, four types of fingerprints are developed, known as the RSSI branch, RSRP branch, united branch, and united direct fingerprint, to explore their contribution to positioning performance. The final proposed approach combines the RSSI, RSRP, and RSRQ into a signal image to form the united direct fingerprint database, therefore Resnet can extract more reliable features for positioning. Additionally, inspired by the idea of transfer learning, feed-forward neural network (FFNN)-based transfer learning uses the prior knowledge of the trained Resnet to achieve better positioning performance. On the other hand, the measurements used in this work are orientation-free, thus there is no requirement for the angle of the smartphone when collecting signals. In order to extract enough features from the fingerprint image dataset, the DNN model was fully trained and tested after each training epoch, and the best performance model was picked as the final model. Additionally, by using cross-entropy loss, dynamically adjusting the learning rate (Lr), and using several helpful data enhancement methods, such as size transformation and random crop, the learning ability of the proposed DNN was further improved.

In conclusion, with respect to the previous work, the main contributions of this paper can be summarized as follows: LTE signal measurements were converted into fingerprint grayscale images for the input of the proposed DNN. In order to find the best positioning fingerprint and explore the contributions of RSSI, RSRP, and RSRQ to positioning performance, four kinds of fingerprint methods were developed. Finally, a hierarchical structure positioning system was designed based on the Resnet-based coarse localizer and transfer-learning-based fine localizer. The proposed positioning approach leverages the pretraining model and fine-tuning model to fully learn the features of the collected fingerprint dataset. Considering the instability of LTE signals and numerous classification points, enough training methods and several data enhancement methods were used to prevent the overfitting problem. The system was tested in a real outdoor environment containing simple and complex outdoor scenes. The experimental results convincingly reveal that the proposed positioning system can reach satisfactory positioning accuracy in outdoor environments.

The rest of the paper is arranged as follows: Section 2 describes the related works. The background and hypotheses are introduced in Section 3. The proposed positioning system is presented in Section 4. In Section 5 and Section 6, the DNN training and positioning methods are described in detail. Section 7 describes the fingerprint classification. The experimental part is described in Section 8. Finally, Section 9 describes the conclusions and future works.

## 2. Related Work

Fingerprint-based positioning technologies can be divided into two main categories: infrastructure-free and infrastructure-based approaches [19]. Infrastructure-free approaches rely on utilizing existing infrastructure, such as CDMA2000, LTE, Frequency Modulation (FM), Wi-Fi, and sound signals, for positioning purposes [20]. On the other hand, infrastructure-based fingerprint positioning technologies focus on dedicated infrastructure, such as Bluetooth, radio frequency identification (RFID) or visible light for localization [21].

In this work, the infrastructure-free approach was adopted, meaning there was no requirement to leverage expensive hardware. The basic idea of fingerprint-based positioning technology is to discover the position of a smartphone device by comparing its signal pattern received from LTE BSs with a pretrained fingerprint database of the signal pattern [22]. Generally, the signal pattern is generated by receiving the RSSI, RSRP, and RSRQ of LTE signals. The fingerprint database is constructed in the offline phase, and contains the RSSI, RSRP, and RSRQ from LTE BSs at known locations.

In order to implement fingerprint positioning, the first step is to establish the fingerprint database [23]. In general, the positioning problem will be transformed into a classification problem. Therefore, places of interest are divided into multiple grids, and each grid is regarded as one class ref. [24]. When constructing a fingerprint database, one person holding a collecting device walks around the grids and collects wireless signals. After completing the task of collecting signals, the fingerprint database, consisting of geotagged signals, can be constructed. Fingerprints can also be constructed by leveraging self-guided robots carrying signal-receiving sensors and automatically roaming around to collect wireless signals, however this approach is currently not a globally economic method [25]. Crowdsourcing relies on volunteers willing to participate in data collection. However, this signal collection method usually takes several days or even a week to complete for large-scale outdoor signal collection work. Therefore, the crowdsourcing method is tedious and time-consuming [26].

The key steps that can affect fingerprint positioning accuracy are the positioning algorithms and the type of fingerprint [27]. The initial shallow models had limited learning ability, leading to unsatisfactory positioning accuracy. With the development of neural networks and computational power, deep learning emerged as a shining star in various fields. Deep learning has excellent learning ability and beats other state-of-the-art approaches in ImageNet competition. Zhang et al. [28] leveraged DNN and a hidden Markov model to locate UE in an indoor and small-scale outdoor area. DelFin, a positioning system using channel state information (CSI), was proposed to realize indoor fingerprint localization [29]. A deep-learning approach utilizing CSI and RSSI to achieve high-precision outdoor positioning was proposed in [30]. Applications of CNN and the use of Wi-Fi and magnetic signals for indoor precise positioning were investigated in [31]. Software-defined radio devices and deep learning approaches were leveraged to improve indoor positioning accuracy [32]. Mondal et al. evaluated the positioning accuracy of a radio fingerprinting algorithm in commercially deployed LTE networks operating on 800 MHz, 1800 MHz, and 2600 MHz frequency bands [33]. Ye et al. utilized unique mapping between the characteristics of a radio channel formulated as a fingerprint vector and a geographic location [34]. The “shape” of the channel frequency response (CFR) could be used to construct a CSI-based fingerprint database. It reduced the memory requirement of the database and the computational complexity of the matching phase [35]. In summary, current deep-learning-based fingerprint positioning approaches focus on developing indoor and small-scale outdoor positioning technologies. Some of these technologies rely on LTE CSI information for positioning. However, these methods rely on dedicated equipment to collect CSI information and, therefore, they are not practical.

Contrary to the aforementioned positioning technologies, the proposed positioning system requires no additional dedicated hardware or specific CSI information. A deep-learning approach is introduced to localize the ubiquitous smartphone.

Compared to other current positioning systems, the proposed system leverages fingerprint-based technology for large-scale outdoor positioning. The proposed approach converts RSSI, RSRP, and RSRQ into fingerprint images for the input of deep learning, and the three fingerprint locations can be sampled at the same time, reducing the collection workload. Ubiquitous LTE signals are utilized, which are easy to collect and free of privacy issues. Additionally, the proposed system requires no orientation information, therefore signals can be collected from any direction. In terms of these aspects, this proposed positioning approach is more practical than other proposed fingerprint-based positioning systems. Table 1 shows a comprehensive list of acronyms for ease of reading.

## 3. Background and Hypothesis

### 3.1. Background for Positioning Parameters

RSRP represents the power value of the subcarrier in the measurement band, which does not contain noise. RSSI represents the average value of the received signal power, which contains other external interference signals. RSRQ is the ratio of cell reference signal power to all signal power values in the cell. Due to the ubiquitous sensors equipped in smartphones, it is now easier to collect RSSI, RSRP, and RSRQ than in the past, when smartphones were not widely used.

**Figure 2:**
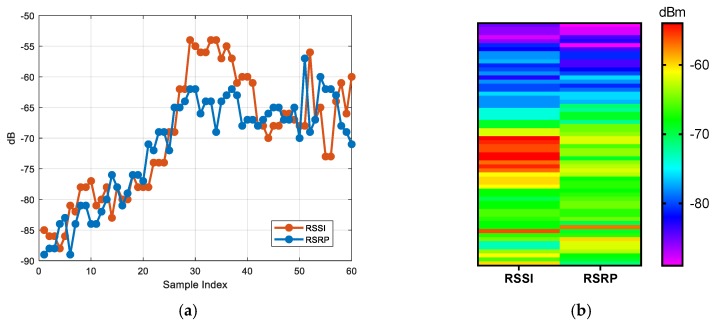
Measurements of RSSI and RSRP at different locations: (**a**) RSSI and RSRP distribution; (**b**) grayscale code of RSSI and RSRP.

Figure 2 shows the changes of RSSI and RSRP in different environments. The RSSIs and RSRPs were collected along a road, and the length of the road was about 200 m; one data point was selected about every 3.5 m and then plotted to form Figure 2. In other words, each sample index in Figure 2 is about 3.5 m apart. This indicates that RSSI and RSRP change with the location, therefore they can be used as features for positioning. However, they may share the same value in different locations. Additionally, as is shown in Figure 1, RSSI and RSRP vary greatly at a fixed position. Therefore, a large set of samples needs to be collected to enable the DNN model to extract differentiated features.

**Figure 3:**
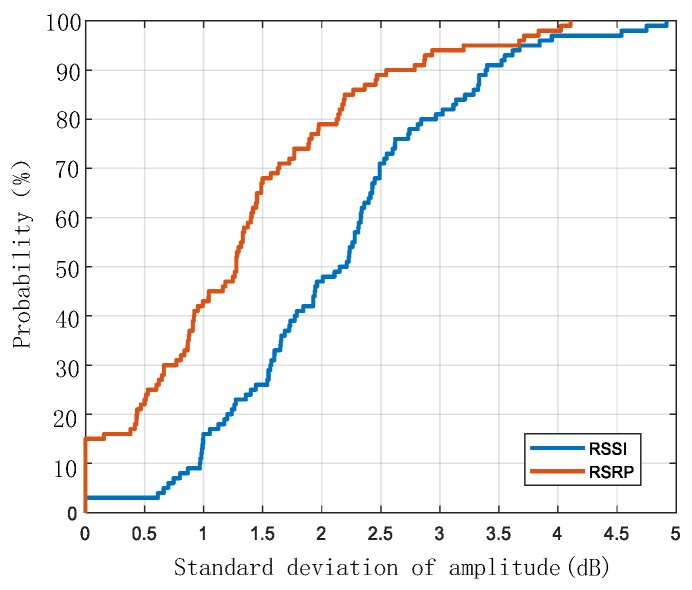
Cumulative distribution function (CDF) of the standard deviations of RSSI and RSRP amplitudes for 100 sampled locations.

Figure 3 shows the cumulative distribution function (CDF) of the standard deviations of RSSI and RSRP values for 100 sampled positions. For all real numbers x, the cumulative distribution function is defined as follows: $F_{X}(x)=P(X\leq x)$. This is the probability that the random variable X is less than or equal to x. For discrete variables, CDF represents the sum of the probability of occurrence of all values less than or equal to x. At each position, 40 to 60 measurements were collected to calculate the value of the standard deviation. Additionally, these measurements were collected in different environments. As shown in Figure 3, for RSRP values, 90% of the standard deviation values are below 2.5. However, for RSSI values, 70% of the standard deviation values are below 2.5. Therefore, RSRP is more stable than RSSI. In the experimental part, these two features are used to locate the UE separately.

### 3.2. Hypothesis 1

Figure 3 shows that compared with RSSI values, RSRP values are more stable at fixed positions, therefore the first hypothesis is that using RSRP as the positioning parameter will achieve better positioning performance. In the experimental parts, these two positioning parameters will be compared.

### 3.3. Hypothesis 2

Different positioning methods will result in different positioning performance. In order to find the best positioning model, in this paper two fingerprint positioning methods are proposed: the united branch method and the united direct method. When using the united branch method for positioning, first the proposed Resnet is used to train with the RSSI branch dataset containing RSSI information and the RSSI branch dataset containing RSRP information, separately. Then, the output feature vectors are extracted and fused together. Finally, multilayer perceptron (MLP) is used to train with the fused features and obtain the positioning model. For the united direct method, the proposed Resnet is directly used to train with the united fingerprint dataset, containing RSSI and RSRP information, and obtain a positioning model. These two positioning methods may have different positioning performance. The second hypothesis is that for the united branch method, the DNN may not effectively extract and utilize the features. Therefore, the united direct method may achieve better positioning performance.

### 3.4. Hypothesis 3

The general definition of transfer learning is storing knowledge gained while solving one problem and applying it to a different but related problem. However, transfer learning is not available in all situations. When transfer learning is designed improperly, its performance may even be weaker. Therefore, the third hypothesis is that transfer learning can be used to improve the performance of DNN in localization scenarios. In the experimental parts, the impact of transfer learning on positioning performance will be explored.

## 4. Proposed Positioning System Architecture

**Figure 4:**
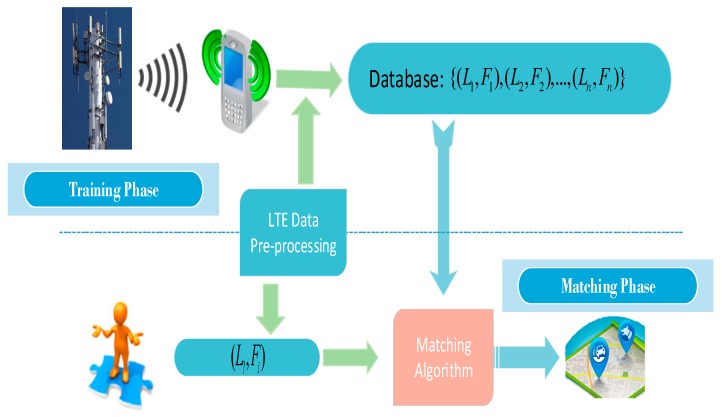
Illustration of proposed outdoor fingerprint-based positioning system.

In this paper, the LTE signal positioning environment consists of multiple LTE BSs. A smartphone equipped with sensors can receive RSSI, RSRP, and RSRQ from the surrounding LTE BSs. The positioning issue in this paper is to estimate the positioning $L_{?}$ of the smartphone device from RSSI, RSRP, and RSRQ observations. As shown in Figure 4, the proposed outdoor positioning system consists of four parts: LTE signal collection, LTE data preprocessing, training phase, and matching phase.

### 4.1. Long-Term Evolution (LTE) Signal Collection

In order to turn the positioning problem into a classification problem, first the area of interest was divided into multiple grids. Each grid corresponds to a category. The grid size is the actual area size corresponding to each category. Then, one person walked around in each predivided grid to collect the LTE signal measurements. After this, in each grid, a series of fingerprint images based on the collected signal measurements was generated. The grid size largely determined the positioning accuracy, and therefore the grid size would not be too large [24].

### 4.2. LTE Data Preprocessing

Thanks to the great achievements of deep learning in the field of computer vision, in this paper measurements of LTE signals are converted into images and computer vision is leveraged to extract signal features. An ordinary RGB image consists of a three-channel matrix: red, green, and blue. The values in the matrix are between 0 and 255. The raw values of RSSI and RSRQ are between −40 and −140 dBm. Therefore, the signal values in this paper were modified based on $v_{s}=(RSSI+150)$ and $v_{p}=(RSRP+150)$. The value of RSRQ does not change much in a small area, and in order to reflect the overall RSRQ at each grid, its value was modified as follows:

(1) $$v_{q}=\sqrt{\frac{1}{N}\sum_{i=1}^{N}{\left(RSRQ_{i}-\bar{RSRQ}\right)}^{2}}$$

where $\bar{RSRQ}=\frac{1}{N}\sum_{i=1}^{N}RSRQ_{i}$, $RSRQ_{i}$ is the collected data in each grid, and N is the data length in each grid.

After modifying the measurements of the LTE signals, three-column vectors consisting of RSSI, RSRP, and RSRQ were leveraged to form the fingerprint grayscale image dataset. For the convenient use of fingerprint images for DNN input, the dimensions of the three-channel matrix of the fingerprint image should be consistent. In each grid, the collected dataset was divided into multiple sub-datasets, then the fingerprint image was built based on these sub-datasets. One way to build a grayscale image is to make the three-channel matrix the same. For example, P stands for the red channel matrix. P is also used to form the green and blue channel matrices to generate the grayscale image. One of the channel matrices can be constructed as follows:

(2) $$\varphi^{i}={\left[v_{s1}^{i},v_{s2}^{i},\ldots ,v_{s30}^{i}\right]}^{T}$$

(3) $$\psi^{i}={\left[v_{p1}^{i},v_{p2}^{i},\ldots ,v_{p30}^{i}\right]}^{T}$$

(4) $$\gamma^{i}={\left[v_{q1}^{i},v_{q2}^{i},\ldots ,v_{q30}^{i}\right]}^{T}$$

(5) $$F_{1}=[\varphi^{1},\gamma^{1},\varphi^{2},\gamma^{2},\ldots ,\varphi^{n},\gamma^{n}]$$

(6) $$F_{2}=[\psi^{1},\gamma^{1},\psi^{2},\gamma^{2},\ldots ,\psi^{n},\gamma^{n}]$$

(7) $$F=[\varphi^{1},\psi^{1},\gamma^{1},\varphi^{2},\psi^{2},\gamma^{2},\ldots ,\varphi^{n},\psi^{n},\gamma^{n}]$$

where $\varphi^{i}$, $\psi^{i}$, and $\gamma^{i}$ are the modified RSSI, RSRP, and RSRQ, respectively, from the *i*th LTE BS, and 30 is the length of each sub-dataset. $F_{1}$, $F_{2}$, and F represent the channel matrices and are used to form the RSSI branch, RSRP branch, and united direct fingerprint image database, respectively. For example, the united fingerprint database can be constructed as follows: $F_{red}=F_{blue}=F_{green}=F$, where $F_{red}$, $F_{blue}$, and $F_{green}$ are the red, blue, and green channels, respectively. The grayscale image can be formed by leveraging these three identical matrices. After constructing the three channel matrices, a fingerprint grayscale image can be formed.

### 4.3. Deep Neural Network (DNN) Training

In order to study the influence of different features on positioning accuracy, different features and united features were used for positioning and the positioning results were compared. After comparing the positioning accuracy of these features, a two-step learning strategy was proposed for training the network. After training was complete, the positioning model was obtained by retaining the model parameters. The main challenge of developing a positioning system is random signal fluctuation. Therefore, the learning ability of the proposed neural networks needed to be strengthened. Additionally, several practical data enhancement methods were adopted.

### 4.4. DNN Positioning

The Resnet algorithm was proposed to compute the UE locations. When using probabilistic methods for positioning, the obtained result will be a probability position estimate. In this paper, probabilistic methods are used for positioning. The outputs of the localizer are the probabilities of all grid-based positions. In Section 6, four positioning methods are proposed and their positioning performance is compared. Then, the best coarse localizer is picked up from the experimental comparison. In order to further increase the positioning accuracy, a transfer-learning-based fine localizer is used to fine-tune the coarse localizer and get better positioning performance. Additionally, it is worth mentioning that one LTE BS covers a certain area, and the approximate location of UE can be determined through the BS to which the UE is connected.

## 5. DNN Training

Different positioning features have a great influence on positioning results. Therefore, the RSSI branch, RSRQ branch, and united branch were separately used as the input of Resnet for positioning. Two united branch strategies were adopted for positioning: direct and branch methods. Then, the positioning accuracy of these features was compared. By leveraging the direct method, a two-step training approach was used to achieve the best positioning accuracy. First, the united fingerprint images were used as the input of the proposed Resnet and achieved a probability result. After obtaining the best positioning accuracy, the parameters of the pretrained model were saved. Then, inspired by the idea of transfer learning, a feed-forward neural network (FFNN) was added after the pretrained model to further improve the positioning accuracy.

In order to reduce the impact of adverse factors, such as gradient dispersion, several data enhancement approaches were adopted. First, the fingerprint images were standardized to 224 × 224 before the input of the proposed Resnet, which made the image features more prominent. Second, the fingerprint was enlarged by 1.25 times and randomly rotated 15°. The batch normalization layer was added after each convolution layer, and a momentum item was added in the batch normalization, which accelerated the convergence of the network and made the training process more stable. These approaches further improved the learning ability of the proposed DNN and improved positioning accuracy.

The number of training epochs has a great influence on the performance of the DNN. When overfitting occurs, learning is fine but the generalization is poor, i.e., learning ability seems to improve, but unfortunately this is done at the expense of generalization performance. Validation is used to avoid this situation. In the training process, first, the fingerprint image database was randomly disrupted, and the first 60% of the dataset was used for training, while the middle 20% and last 20% were used for validation and testing. For each training epoch, the accuracy of the test set was tested, then the positioning accuracy and the parameters of the trained model were reserved. After completing the training section, the model with the best positioning accuracy was used as the final model.

The learning rate is a hyperparameter that guides us on how to adjust the weight of the network through the gradient of the loss function. Learning rates that are too small will cause a slow gradient descent, and may cause the network to converge to a local minimum. Conversely, a large learning rate will cause the network gradient to drop too quickly and cause it to oscillate back and forth. Therefore, in order to effectively alleviate the occurrence of overfitting and achieve a regularization effect, the learning rate needs to be dynamically adjusted during the training process. Every 35 epochs the network is trained, the learning rate is automatically adjusted to half of the original size.

## 6. DNN Positioning

The proposed positioning system consists of the location features of the RSSI, RSRP, and RSRQ fingerprint using a Resnet. In this section, the proposed algorithm architecture and models are introduced. For the proposed positioning system, the contributions of RSSI, RSRP, and RSRQ to the positioning performance will be analyzed. Finally, the final design of the positioning architecture is presented.

### 6.1. Deep Residual Network Introduction

Convolutional neural networks have made great contributions in many fields, especially in the field of computer vision. Many studies have shown that as the DNN deepens, its learning ability will increase. However, a very deep CNN will face the problem of gradient diffusion, which will deteriorate the learning ability of the CNN model. Earlier studies tried to adopt initialization methods and layer-wise training to solve this problem. Additionally, the rectified linear unit (ReLU), scaled exponential linear unit (SELU), and the ReLU activation function have been used to prevent the gradient dispersion problem. Batch normalization standardizes the mean and variance of hidden layers for each minibatch. However, not all networks are easy to optimize. He et al. [33] proposed Resnet to solve this problem, which leverages a simple connection mechanism to propagate information to deeper layers of networks. It has effectively proved its excellent learning ability in ImageNet competitions.

**Figure 5:**
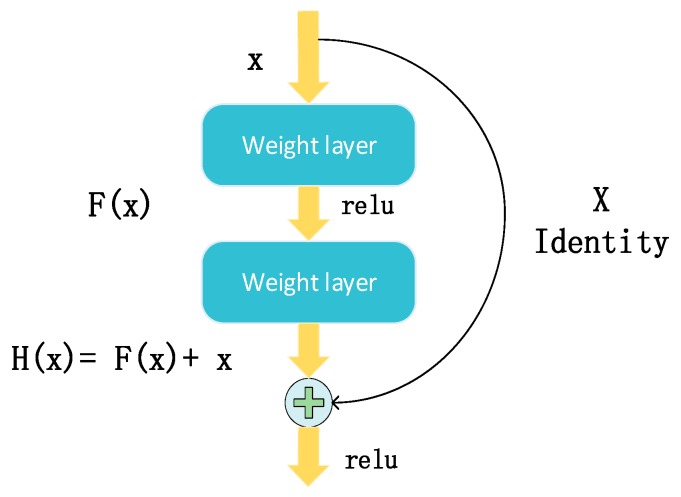
Residual unit structure diagram. ReLU, rectified linear unit.

The basic idea of Resnet is that the target network fits H(x), and the original mapping is F(x)+x. When H(x) is too complex to learn, Resnet will change the non-linear mapping F(x) to 0 and skip blocks of the weight layer by leveraging shortcut connections to form residual blocks [36]. Such residual blocks have successfully solved the gradient dispersion problems occurring in other DNNs. The basic blocks of Resnet are shown in Figure 5.

**Figure 6:**
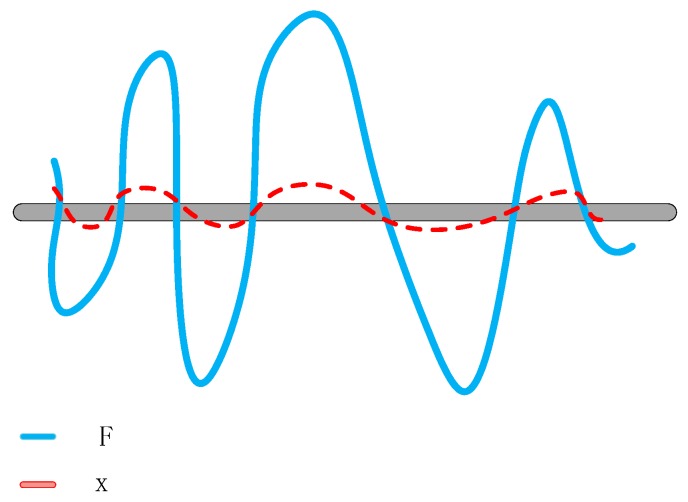
Deep residual network (Resnet) transfers the learning problem from reaching F (blue line) to reaching the direct linear mapping x (red line).

As shown in Figure 6, Resnet transfers the learning problem to the residual mapping, which is simpler and easier to learn. Resnet creates several direct paths for propagating gradient information between these residual blocks. Therefore, layers in the upper residual blocks can propagate information directly to layers in lower residual blocks.

**Figure 7:**
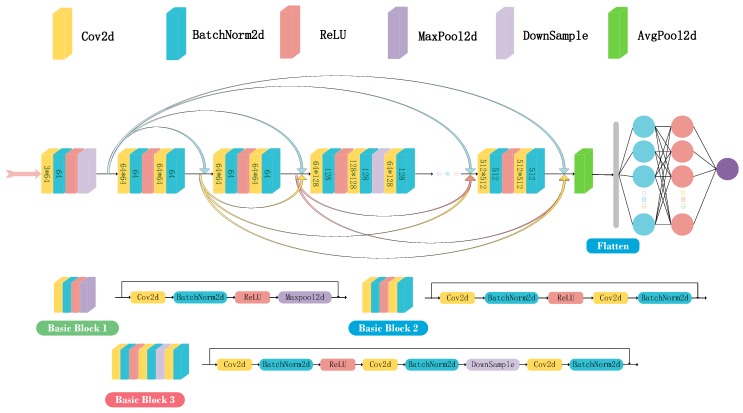
Overall architecture of proposed Resnet.

As shown in Figure 7, the proposed Resnet consists of one basic block 1, four basic block 2’s, three basic block 3’s, one average pooling layer, and two hidden multilayer perceptrons (MLPs). The convolution 2D, batch normalization, ReLU activation function, max-pooling layer, and downsample layer are leveraged to form these three basic blocks. Additionally, each basic block can be regarded as a residual block. When a problem such as gradient dispersion occurs during the training process, by leveraging shortcut connections, the network can skip these basic blocks and continue training.

For the convolution layer, the padding step is first used to extend the input tensor size by adding some zero elements at two dimensions. In the proposed positioning system, (3,3) zero-padding is added in basic 1, and (1,1) zero-padding is added in basic 2 and 3.

Then, the convolution step slides through the entire image step by step. The output of the convolution step is defined by the number of convolutional kernels K, the kernel size $\left(U_{1},U_{2}\right)$, the *k*th convolutional kernel $W_{k}=(w_{u_{1},u_{2},t}^{k})\in {\mathbb{R}}^{U_{1}\times U_{2}\times T}$, and the strides of the convolution window $(a_{1},a_{2})\in {\mathbb{N}}^{2}$.

Batch normalization is added to prevent the problem of degradation and accelerate the convergence speed. The ReLU activation function is used to proceed with the input tensor of the previous layer.

For basic block 1, the new tensor becomes the input of the max-pooling layer. For basic block 2, the convolution layer and batch normalization layer follow, and the processing mechanism is the same as in the above methods. For basic block 3, the downsample layer is added after batch normalization. After propagation of these basic blocks, the average pooling layer is added to downsample the input tensors. The mechanisms of average pooling and downsampling are the same as for max pooling.

Normally, several fully connected layers are added to further increase the learning ability of the neural network.

Then, the SELU activation function is used to proceed with the output value of every fully connected layer.

Finally, softmax is used to predict the positioning accuracy. When optimizing the DNN structure, the cross-entropy loss is used to update its parameters. The specific parameter calculation process of each layer can be obtained from [17,29].

### 6.2. Received Signal Strength Indicator (RSSI) and Reference Signal Receiving Power (RSRP) Branches

In order to study the influence of RSSI and RSRP on the positioning performance, $F_{1}$ and $F_{2}$ were used to form the RSSI branch and RSRP branch fingerprint grayscale image dataset. The proposed positioning Resnet leveraged the RSRI and RSRP branches to separately learn the high-level location features. Then, Resnet was used to learn the relationship between the ground-truth location and the high-level features to predict the location.

The proposed Resnet learns the fingerprint image features by adjusting the weights and bias of the network. The convolution matrix is randomly initialized and slides along the fingerprint images to extract the features. For each training epoch, a new fingerprint feature map is generated to represent the feature map. The network leverages cross-entropy loss and adaptive moment estimation (Adam) algorithms to update the weights and bias.

### 6.3. United Branch

**Figure 8:**
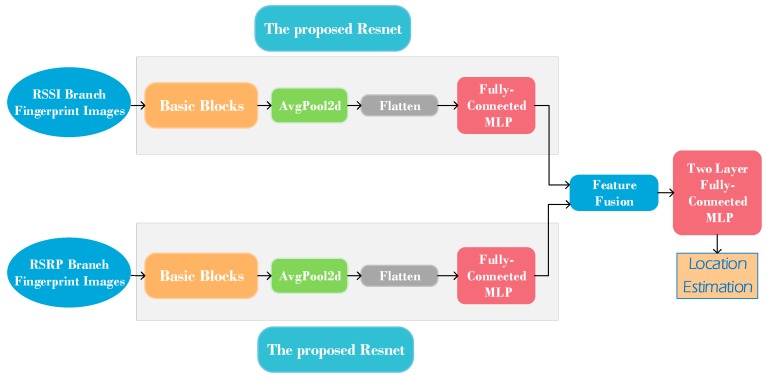
Architecture of proposed united branch positioning method.

In order to take advantage of the RSSI and RSRP fingerprint images, this work compares two united positioning approaches: united branch and united direct methods. The united branch method is as follows: first, Resnet is used to train with the RSSI and RSRP branches, then the output feature vectors of Resnet are extracted and these two feature vectors are fused together, as shown in Figure 8. Then, a fully connected MLP is used to train the fused feature and achieve probability positioning results. The united direct method leverages F to form the united direct fingerprint dataset, then Resnet is used to train with the united fingerprint dataset and obtain a location estimation. Therefore, this method directly combines RSSI, RSRP, and RSRQ information for localization.

**Figure 9:**
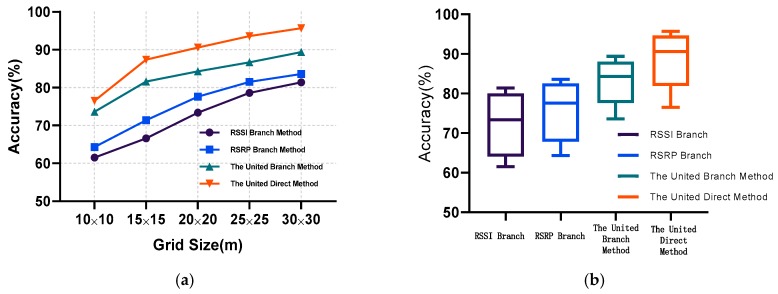
Performance comparison of different localization methods: (**a**) positioning performance of methods with respect to different grid sizes; (**b**) average and range of positioning accuracy of methods over all grid sizes.

Figure 9 shows the impact of different positioning methods and grid sizes on positioning accuracy. Different grid sizes correspond to different positioning datasets. When collecting data, the positioning area was divided into multiple 5 × 5 m grids and LTE signal measurements were collected from each grid. Then the 5 × 5 m grid was used to form other grids. For each method, the positioning accuracy was compared at different grid sizes. The experiments were conducted three to five times and the best accuracy was picked as the final accuracy. The RSRP branch method achieved higher positioning accuracy than the RSSI branch method. Owing to the instability of RSSI, the proposed Resnet was not able to effectively extract reliable features, therefore the RSSI branch method obtained the worst positioning accuracy, which validates hypothesis 1. Moreover, different training methods can make a difference in positioning performance. The united branch method showed better positioning performance than RSSI branch and RSRP branch positioning methods. Because the fully connected MLP was not able to simultaneously capture features from the RSSI and RSRP branches, the united branch method achieved the second-best positioning performance, The united direct method achieved the best positioning performance, which was trained as the proposed coarse localizer.

**Figure 10:**
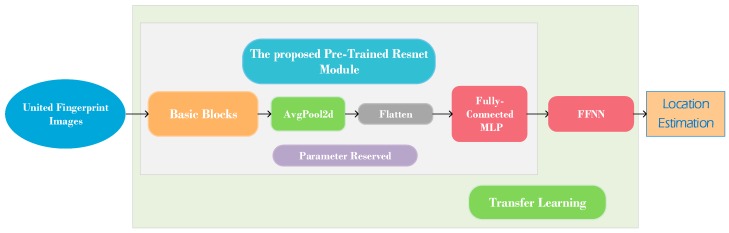
Architecture of proposed DNN positioning method. The positioning system consists of two parts: Resnet-based localizer and feed-forward neural network (FFNN)-based fine localizer.

Generally, transfer learning is defined as the application of knowledge or patterns learned in a task to different but related fields or problems [37]. Inspired by the idea of transfer learning, in this paper a transfer-learning-based fine localizer was developed, and the idea of transfer learning is reflected in using the prior knowledge of the pretrained model and applying it to the new customized FFNN model. Specifically, as shown in Figure 10, first the united direct fingerprint images were used as the input of the proposed Resnet. After completing the training process, the model with the best positioning accuracy was obtained, and then the parameters of the Resnet positioning model were reserved and this model was regarded as the pretrained model, called the coarse localizer. Then, the FFNN was added after the pretrained Resnet for transfer learning. This transfer learning method leverages the prior knowledge of the pretrained model to find the patterns between ground-truth locations and the prediction information, and finally estimates the user locations. The new transfer-learning-based model was then used as the fine localizer to achieve better positioning accuracy. Table 2 shows the DNN parameter configuration in detail.

## 7. Fingerprint Classification

The proposed positioning system converted the positioning problem into a classification problem. Therefore, the system divided the area of interest into multiple grids as classification points. The size of the grids largely determined the positioning accuracy. As shown in Figure 9, a small grid will lead to bad positioning performance, and a large grid will lose the meaning of the positioning. Additionally, with a further increase of the grid size, the positioning accuracy did not improve significantly. In order to achieve satisfactory positioning, the classification points should be dense enough; therefore, the grid size was set to 15 × 15 m.

In this study, one person walked around the grids and held a smartphone to collect the LTE signal measurements. Then, the raw LTE signal samples were classified into different classification points according to the preset grid size. All fingerprint images were required to have the same size.

## 8. Experiment and Evaluation

**Figure 11:**
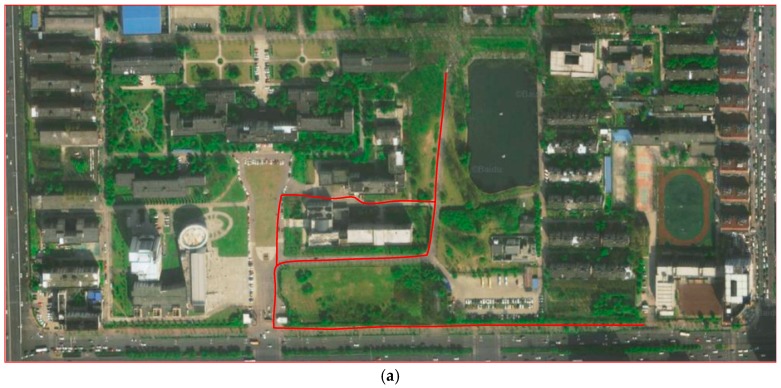

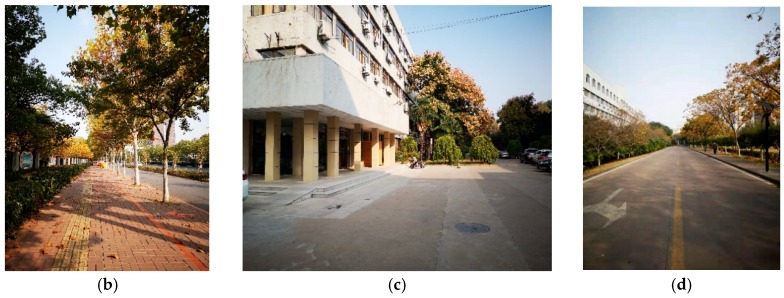
(**a**) Satellite map and (**b**–**d**) photographs of outdoor positioning area.

In order to validate the usability of the proposed positioning system, extensive experiments were conducted in a real outdoor environment containing various scenes. Figure 11 shows the layout of the area of the experiment. It did not require real positions of the LTE BSs. LTE signal measurements are influenced by trees, moving cars, and people. Considering the variety of scenes, the experimental area was suitable for the evaluation of the proposed positioning system.

The total length of the experimentally followed paths was approximately 1050 m; 15 × 15 m was selected as the grid size for the experiments. When collecting the raw LTE signal information, all test areas were discretized into dozens of grids, and we held a smartphone and walked around in each predivided grid to collect signal measurements. By using the Cellular-Z application, RSSI, RSRP, and RSRQ could be sampled at the same time [38].

The positioning system was implemented on a Dell PC with an RTX2060 graphics card; this has powerful data processing compared to smartphone platforms. The proposed positioning models, data preprocessing, and data enhancement methods were implemented on Pytorch. The key parameters used in the proposed DNN-based positioning algorithm were evaluated. Additionally, the proposed method was compared with other state-of-the-art positioning methods.

### 8.1. Influence of Feed-Forward Neural Network (FFNN)-Based Transfer Learning

**Figure 12:**
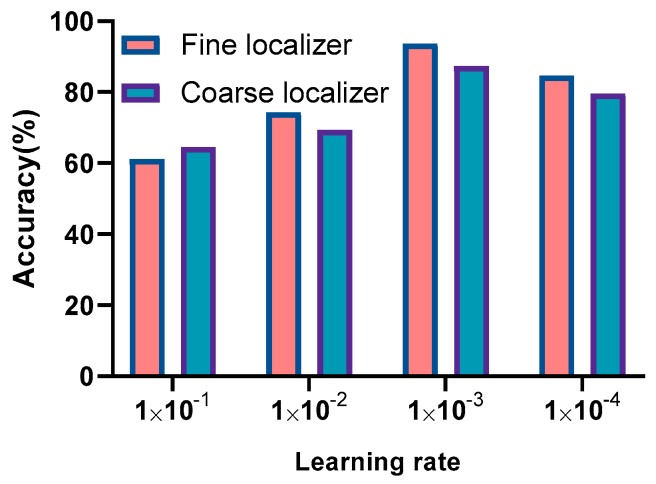
Positioning performance of Resnet-based coarse localizer and transfer-learning-based fine localizer with different initial Lr values.

In this experiment, the influence of FFNN-based transfer learning was evaluated for positioning performance. Each learning rate experiment was conducted three to five times and the best accuracy was picked as the final accuracy. The positioning performance was also separately tested with different initial Lr values of fully connected MLP and FFNN. Figure 12 shows that the transfer-learning-based fine localizer generally achieved better positioning performance compared with the Resnet-based coarse localizer. When the initial learning rate was large, the positioning accuracy deteriorated greatly. This is because a large learning rate leads to the network skipping the minimum point during the training process. A high learning rate may cause the loss to continue to increase, which conflicts with the goal of improving the model’s learning ability. A small learning rate causes the network to converge to a local minimum, which deteriorates the network’s learning ability. Therefore, the learning rate should be neither too large nor too small.

### 8.2. Influence of Different Activation Functions

**Figure 13:**
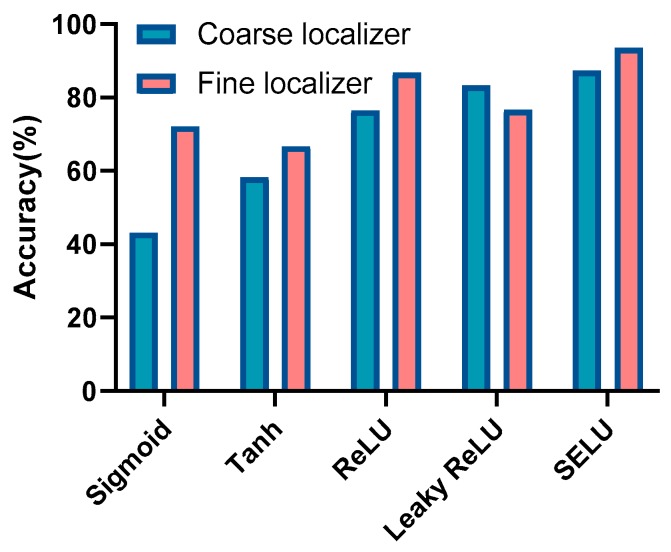
Positioning performance with different activation functions.

The activation function can play a role in mapping the data features to the new feature space, which can be more conducive to data training and speed up the convergence of the model. The commonly used activation functions are sigmoid, Tanh, ReLU, leaky ReLU, and SELU. When neural networks began to emerge, the most commonly used activation function was sigmoid. However, it tends to have a gradient-vanishing problem and its operation is time-consuming. The Tanh activation function came along to solve the problem of sigmoid. Currently, the most widely used activation function is ReLU, which converges faster and has lower computational complexity than the others. However, it has dead ReLU problems: some neurons may never be activated, causing the corresponding parameters to be unable to be updated. Leaky ReLU is used to further solve the problem of ReLU. Finally, the SELU activation function was proposed, which has the advantages of leaky ReLU and is more stable. As shown in Figure 13, owing to the excellent modeling ability, the SELU activation function achieves the best positioning performance. In most cases, the transfer-learning-based fine localizer performs better than the coarse localizer.

### 8.3. Influence of the Number of Hidden Layers on the Fully Connected Multilayer Perceptron (MLP)

**Figure 14:**
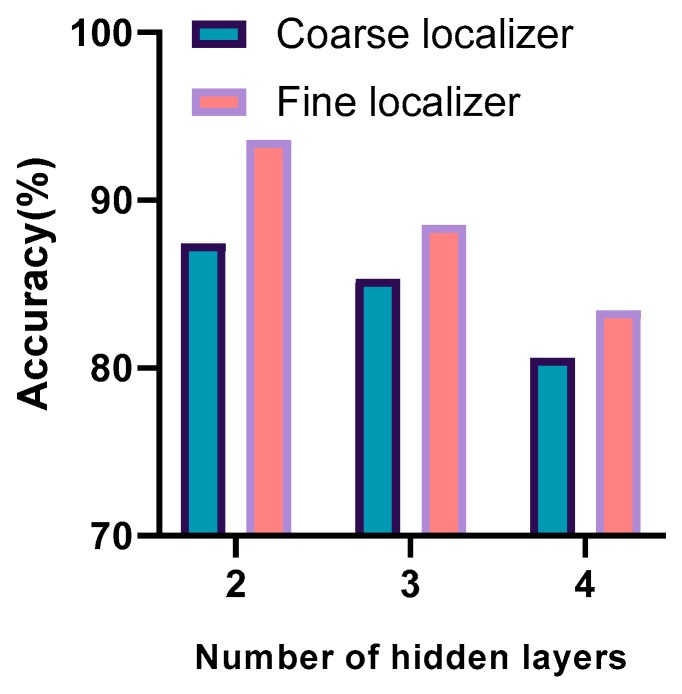
Positioning performance with different numbers of hidden layers.

The number of hidden layers has a great influence on the performance of the DNN. Figure 14 shows that as the number of hidden layers increases, the positioning performance deteriorates. It can be concluded that complexity does not give a better location estimation; this is because the complexity of the network structure will cause the network to fall into a local minimum during the training process.

### 8.4. Performance with Different Batch Sizes

**Figure 15:**
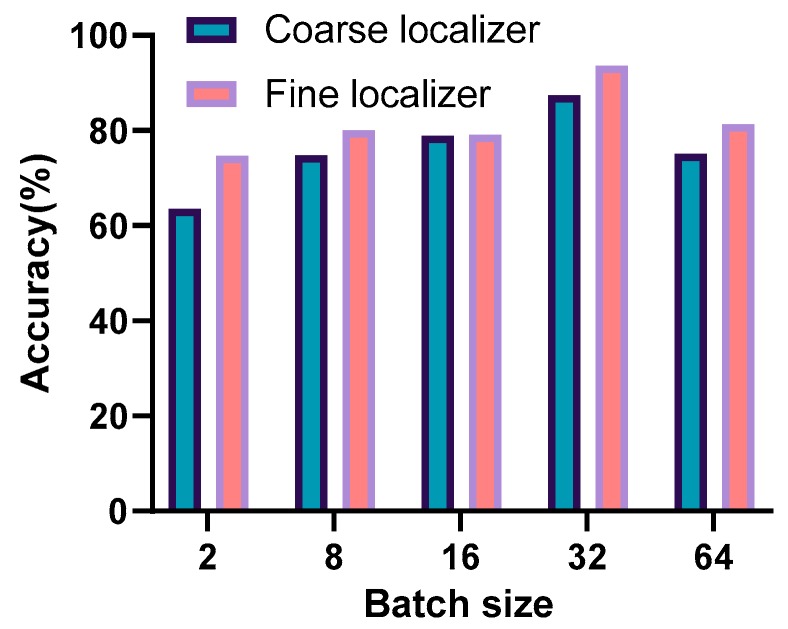
Positioning performance with different batch sizes.

Generally, the larger the batch size, the more accurate the descent direction and the smaller the oscillation. If the batch size is too large, a local optimum may occur. A small batch size will cause network training to be more random and it will be difficult to achieve convergence. Each batch size experiment was conducted three to five times and the best accuracy was picked as the final accuracy. As shown in Figure 15, the best performance was observed when the number of BSs was 32. Figure 12, Figure 13, Figure 14 and Figure 15 indicate that in most cases, the transfer learning-based fine localizer shows better positioning performance than the coarse localizer. However, when some parameter settings are inappropriate, its performance is not as good.

### 8.5. Performance Comparision

**Figure 16:**
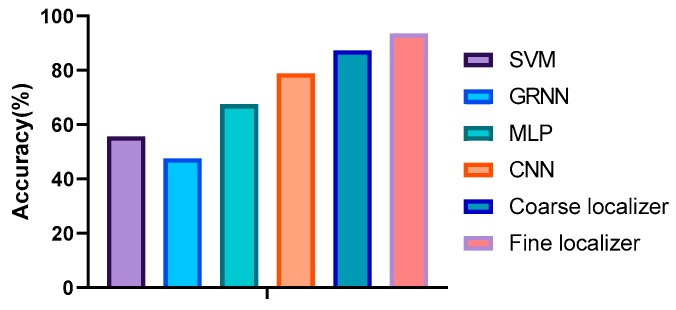
Positioning performance with different algorithms.

In order to evaluate the performance of the proposed algorithm, the positioning performance with other classic algorithms was compared. Each comparative experiment was conducted three to five times and the best accuracy was picked as the final accuracy. Since many SVM-, GRNN-, and MLP-based positioning techniques use raw signal measurement data for positioning [10,14,15], in order to compare the positioning accuracy of the proposed method with the commonly used methods raw LTE signal measurements were used for the input of SVM, GRNN, and MLP, and the proposed fingerprint image dataset as the input of CNN, the coarse localizer, and the fine localizer. It is worth mentioning that the fingerprint image dataset was formed by the raw signal measurement data. When using multiclass SVM for positioning, the Gaussian kernel is used as the kernel function, with the kernel scale set to sqrt(P)/4, where P is the number of predictors. For the GRNN, we set its smoothing factor to 1. For SVM and GRNN, 80% of the dataset was used for training and the remaining 20% for prediction. Neither were optimized. When using MLP for positioning, the number of hidden layers was set to 3, and each hidden layer contained 200 neurons. The SELU function was used as the activation function. Figure 16 shows that, compared to other learning algorithms, the proposed algorithm achieved the best positioning performance. This is because shallow algorithms like SVM and GRNN have limited learning ability. Conversely, owing to the powerful computing power and excellent learning ability, deep models like MLP and the proposed models can extract reliable features and outperform other algorithms.

## 9. Conclusions

This paper proposes a grayscale fingerprint image-based outdoor positioning method. By leveraging a deep learning algorithm, it can find the position from the fingerprint database that best matches the real UE position. This is a novel grayscale fingerprint representation approach to convert LTE signal measurements into grayscale fingerprint images, which are used to construct a fingerprint database. Then, these images are used as the input of the proposed DNN and support achieving satisfactory positioning performance. Three hypotheses were proposed to guide the research. In order to figure out the contributions of the different LTE signal measurements, the positioning system was tested considering both the RSSI and RSRP branches. To obtain the best-performing model, the united branch and united direct positioning methods were proposed, and the united direct fingerprint was used to construct the fingerprint database, then it was used to obtain the coarse localizer. To overcome the instability of the LTE signal, the proposed DNN was fully trained and the best-performing model was picked out as the final model. Additionally, several data enhancement methods were leveraged to improve the robustness of the positioning system. Finally, the superior performance of the proposed positioning system was validated by the experiments. Owing to the orientation insensitivity of LTE signal measurements and the strong learning ability of the proposed DNN, the proposed positioning system has no restriction on the orientation of UE, and it achieves satisfactory positioning performance compared to other state-of-the-art positioning methods. For future work, we intend to improve the application of the processing methods of the source information (RSSI, RSRP, and RSRQ) in order to reduce the need for processing capacity, streamline the method, and reduce the time needed to obtain the relevant location information. Additionally, we intend to cooperate with a local company to achieve engineering goals and apply this method to the IoT.

## Figures and Tables

**Table 1:** Comprehensive list of acronyms for ease of reading.

Acronym	Definition
DNN	Deep neural network
RSSI	Received signal strength indication
RSRP	Reference signal receiving power
RSRQ	Reference signal receiving quality
Resnet	Deep residual network
MLP	Multilayer perceptron
Lr	Learning rate
CSI	Channel state information
CDF	Cumulative distribution function
OLBS	Outdoor location-based services
AOA	Angle of arrival
TOA	Time of arrival
TDOA	Time difference of arrival
UE	User equipment
BS	Base station
LTE	Long-term evolution
GPS	Global positioning system
NLOS	Non-line of sight
LOS	Line of sight
APs	Access points
KNN	K-nearest neighbors
WKNN	Weighted k-nearest neighbors
GRNN	Generalized regression neural network
SVM	Support vector machine

**Table 2:** Testing configurations for performance evaluation. Adam, adaptive moment estimation; scaled exponential linear unit (SELU), scaled exponential linear unit.

Unit	Parameters	Values
Pretrained Resnet	Activation function	ReLu
	Optimizer	Adam (initial lr = 0.001)
	Loss	Cross-entropy loss
FFNN	Activation function	SELU
	Optimizer	Adam (initial lr = 0.001)
	Loss	Cross-entropy loss
	Dropout	0.5
Output layer	Activation function	Softmax
